# A pregnant women with history of hashimoto's thyroiditis diagnosed with Kikuchi-Fujimoto disease: the first case report

**DOI:** 10.1186/s13044-022-00135-3

**Published:** 2022-09-15

**Authors:** Bashar Bazkke, Joli Osman, Mohammad Shahrour, Mohammad Ziadeh, Aya Haji Mohamad, Mohamed Imad Eddin Mouhandes, Ammar Niazi

**Affiliations:** 1 Faculty of Medicine, University of Aleppo, Aleppo, Syria; 2 Department of Pathology, Faculty of Medicine, University of Aleppo, Aleppo, Syria; 3 Department of General Surgery, Faculty of Medicine, Aleppo University Hospital, Aleppo, Syria

**Keywords:** Kikuchi-Fujimoto disease, Lymphadenitis, Lymphadenopathy, Hashimoto's Thyroiditis, Pregnancy

## Abstract

**Background:**

Kikuchi-Fujimoto disease (KFD) is a benign, self-limiting disorder characterized by regional lymphadenopathy. Clinical symptoms range from mild fever and tenderness to upper respiratory syndrome. A few cases have been observed during pregnancy or Hashimoto's disease. What we describe here is the first observed case of KFD in a pregnant woman with a history of Hashimoto's thyroiditis.

**Case presentation:**

A 36-year-old woman presented to Aleppo University Hospital during the 13^th^ week of gestation with a painful cervical node on the right side of her neck. The patient's previous medical history confirmed Hashimoto's thyroiditis for several years. After histopathological examinations and radiological investigations, she was diagnosed with Kikuchi-Fujimoto disease and treated with corticosteroids. Although the patient did not adhere to the treatment very well due to her concerns for the fetus, the clinical picture improved after delivery. The patient now is on follow-up and continuing the current treatment with corticosteroids.

**Conclusions:**

Further investigations need to be conducted to understand the possible autoimmune etiology of KFD when it is associated with Hashimoto's thyroiditis disease. It is also necessary to understand the relationship between this disease and pregnancy.

## Introduction

Over the last 40 years, there has been a growing interest in the histiocytic necrotizing lymphadenitis disease or Kikuchi-Fujimoto disease (KFD). KFD was named after Kikuchi & Fujimoto who reported the first case in 1972 in Japan [1].

KFD is a benign, unilateral, self-limiting disorder, characterized by regional lymphadenopathy, and usually seen in first three decades in female patients [2].

Here, we report a case of a pregnant woman with unilateral painful cervical nodes, who was diagnosed with KFD and had a prior diagnosis of Hashimoto's thyroiditis.

## Case presentation

**Fig. 1:**
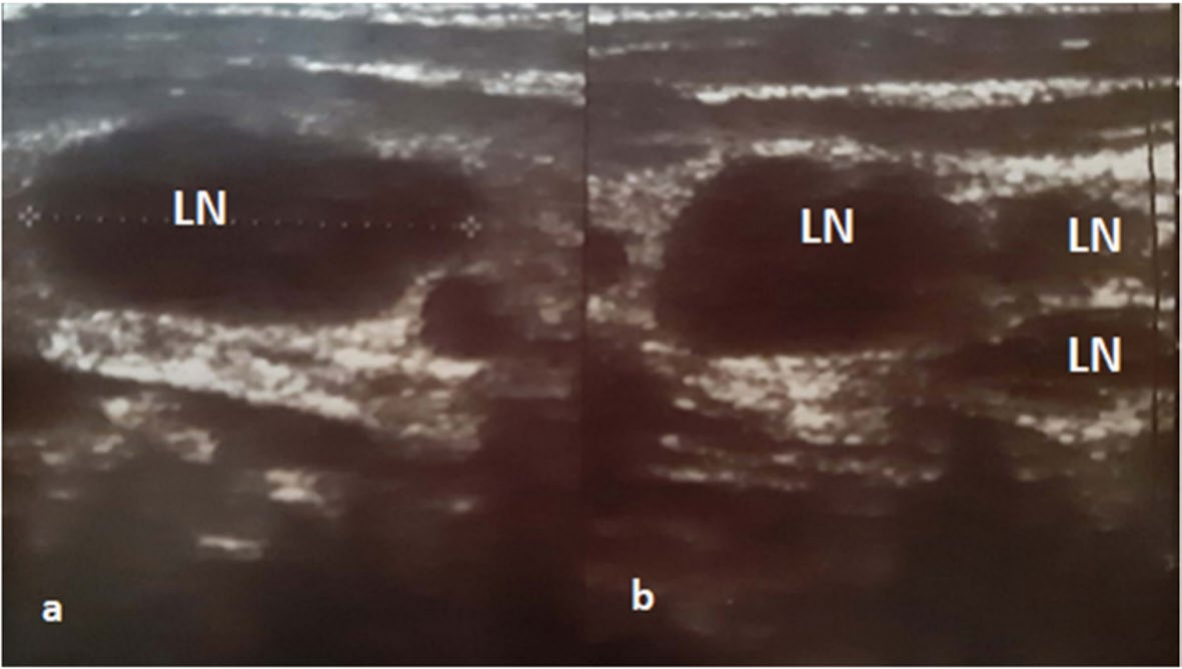
Neck ultrasound prior to delivery. **a** The largest lymph node. **b** Multiple lymph nodes (LN) on the right posterior triangle, the largest one measures 10 × 20 mm with loss of fatty core

A 36-year-old woman, gravida 6 para 6, presented during her 13^th^ week of gestation with a painful enlarged cervical node on the right side of her neck, along with rigors, difficulty in moving her right arm, fatigue, and anorexia. She had a history Hashimoto's thyroiditis (TSH 9.7ulU/ml) for the last 6 years and underwent a C-Sect. 7 years ago. Family history included a father with colon cancer and a mother with Hashimoto's thyroiditis. The physical examination showed enlarged posterior cervical lymph nodes located laterally to the right sternocleidomastoid muscle. Complete blood count (CBC) and chemistry panel were normal. Negative Tuberculin test excluded TB. However, there were elevated levels of anti-toxoplasmosis IgG antibodies 112 IU/ml. The cervical ultrasound revealed right-sided lymph nodes between 10—20 mm (Fig. 1).

**Fig. 2:**
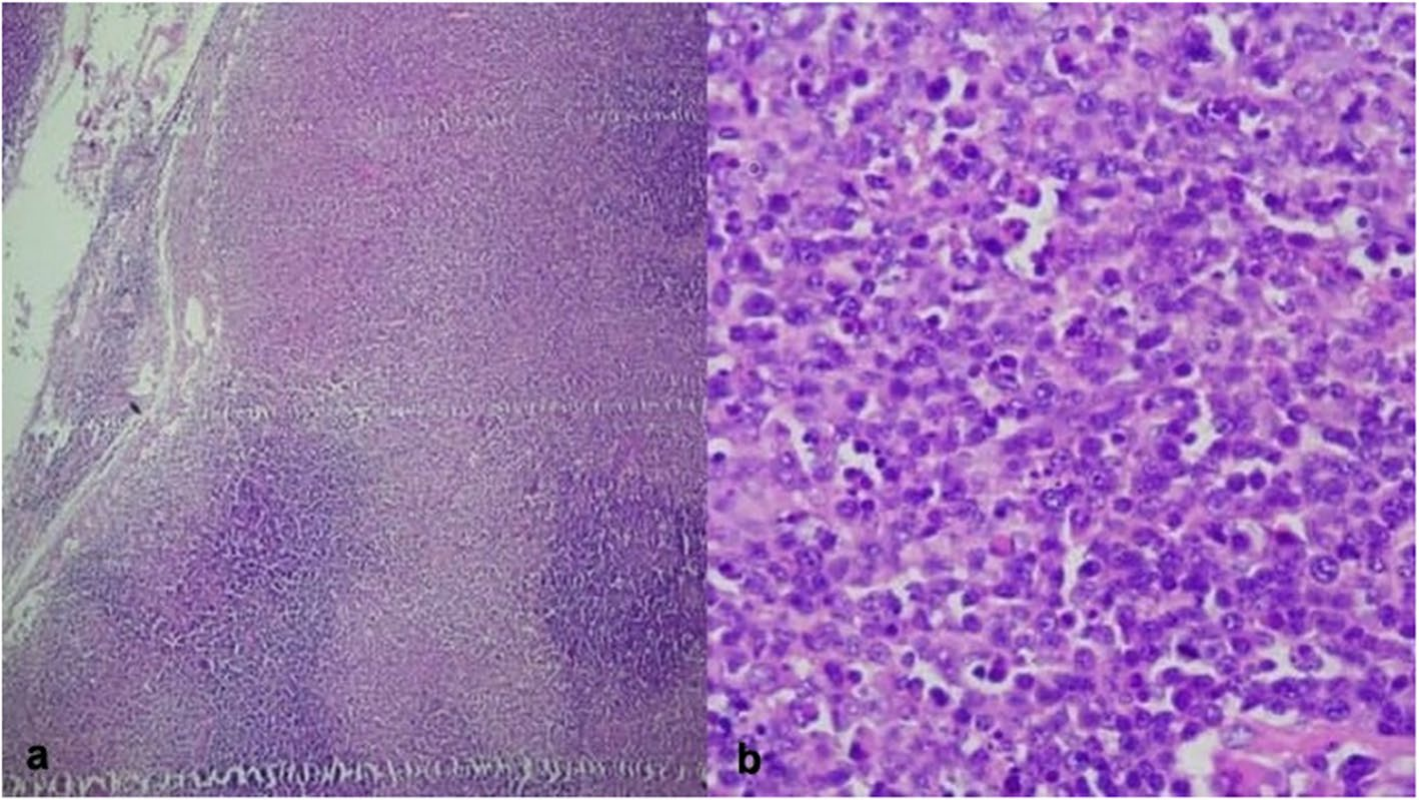
Excisional lymph node biopsy prior delivery. **a** Partially affected architecture, reactive follicles, Paradoxical hyperplasia, and pale wedged-shaped areas of paracortical necrosis. **b** Cellular areas show abundant plasmacytoid dendritic cells and scattered immunoblasts

As the patient was pregnant, Computed Tomography (CT) Scan was contraindicated. Although TB was excluded by lab results and the IgG levels indicated a prior exposure to Toxoplasma, other more serious diseases should be excluded from the differential diagnosis including malignancies especially Hodgkin's lymphoma. Therefore, an excisional cervical node biopsy was performed, and a 2 cm lymph node was obtained for pathological evaluation. As a result, microscopic findings (Fig. 2a, b) demonstrated necrotic areas containing abundant apoptotic debris including crescentic histiocytes, with the absence of neutrophils and heterogeneous mononuclear cells. Overall features suggested Kikuchi–Fujimoto disease.

**Fig. 3:**
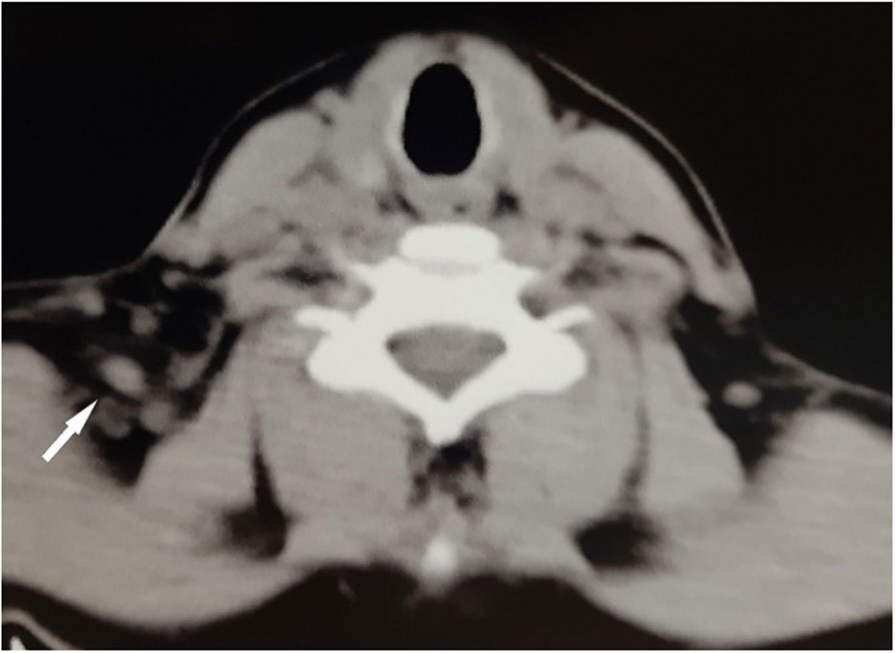
CT scan of the neck after delivery. Multiple lymph nodes (LN) on the right posterior triangle with edematous adipose contour

Our patient received corticosteroids for 20 days, then stopped the treatment on her own, because she was worried about the health of her fetus, despite the doctors' attempts to reassure her that the drug was safe. The patient underwent a C-section without any complications and had a healthy 3000 g male. The clinical picture improved after delivery, but the cervical ultrasound showed no remarkable change. CT scan was preformed after delivery and showed enlarged lymph nodes on the right side of the neck (Fig. 3).

As a result, the patient will continue with corticosteroid treatment for the next two months after which she will be re-evaluated.

## Discussion and conclusions

Kikuchi-Fujimoto disease (KFD), which is also known as histiocytic necrotizing lymphadenitis is a benign and self-limiting disorder, associated with regional lymphadenopathy, tenderness, fever and other classic findings.

Kikuchi and Fujimoto first reported the disease in 1972. It mostly affects females under 40 years and most cases are reported from Asia [2, 3].

The etiology remains unclear, but Kikuchi-Fujimoto Disease has clinical and histologic features similar to some autoimmune diseases and can accompany many autoimmune disorders such as systemic lupus erythematosus (SLE), primary Sjögren syndrome, SLE-like and Hashimoto's disease, thus some reports suggest an autoimmune origin of KFD [1].

Here we report a case of a pregnant woman, who was diagnosed with KFD and had a history of Hashimoto’s thyroiditis disease.

To the best of our knowledge, this is the first case in the literature of a pregnant woman who was diagnosed with KFD with a history of Hashimoto’s disease.

Twelve cases of KFD associated with pregnancy were published in the literature. The majority of these cases presented during the first trimester of pregnancy. In one case the episode started at the 28^th^ week of gestation [4]. In our case, the patient presented at the 13^th^ week of gestation.

KFD mostly presents for the first-time during pregnancy. In 5 cases, recurrent episodes were reported [4–8]. In one case there was a recurrent bout after 24 h from delivery [4].

Fourteen cases in the literature reported KFD in patients with Hashimoto's disease.

However, KFD mostly presents with unilateral cervical lymphadenopathy, more commonly in the posterior triangle, in addition to fever and chills.

More than 59% of patients are seen to have painful lymphadenopathy. Other rare symptoms may present like nausea and vomiting, weight loss, diarrhea, night sweats, hepatosplenomegaly, headache, ataxia, and aseptic meningitis [2].

Our patient presented with unilateral painful lymphadenopathy in the right posterior cervical lymph nodes, which caused difficulty in moving her right arm. She also suffered from tachypnea and anxiety, besides fatigue and anorexia, which may indicate uncontrolled hypothyroidism.

The diagnosis of KFD may be difficult because of the wide differential diagnosis, which includes systemic lupus erythematosus, toxoplasmic lymphadenitis, infectious mononucleosis and cat-scratch disease. But it is essential to differentiate KFD from malignant lymphoma to avoid unnecessary therapy [3].

The gold standard to diagnose KFD is the excisional biopsy. KFD does not have a special appearance on ultrasound or CT [2].

Although there is no specific diagnostic laboratory test for this disease, some tests should be done to exclude diseases like TB or to deny the presence of comorbidities that cause extraordinary symptoms, like SLE. Elevated erythrocyte sedimentation rate (ESR), mild neutropenia, and lymphocytosis could exist in some cases [9].

Our patient was pregnant, so we could not proceed a CT scan before the delivery. An ultrasound was performed during the pregnancy and showed enlarged right posterior cervical triangle lymph nodes.

The histological examination for the cervical node revealed KFD, as it showed a cellular area with abundant plasmacytoid dendritic cells and scattered immunoblasts and pale wedge-shaped areas of paracortica necrosis.

KFD is a self-limiting disease, which requires no specific treatment. However, sometimes there is a need for symptomatic treatment like analgesics, antipyretics, and rest. Patients with severe symptoms or with recurrent disease need further treatment and may benefit from corticosteroids course [2]. If severe manifestations appeared during pregnancy, steroids or hydroxychloroquine can be administered [4]. This patient received corticosteroids but did not adhere to the treatment because she was worried about the fetus health.

The patient underwent caesarean section and delivered a healthy infant weighing 3000 g with no complications. After the delivery another ultrasound was performed and the lymph nodes were still enlarged. A CT-scan without injection was also performed and showed enlarged lymph nodes in the posterior cervical triangle, the carotid triangle, and the supraclavicular area.

From our experience and considering the published cases, KFD has no negative effect on fetuses. Also, pregnancy can induce the disease or trigger a recurrent episode of the disease. Therefore, we recommend further research to explain the relationship between KFD and pregnancy or autoimmune diseases.

Although KFD is usually a self-limiting disease and has no impact on the fetus, early investigations should be made in any pregnant women with cervical lymphadenopathy to exclude more serious diseases from the differential diagnosis and to avoid unnecessary treatment. Further studies should be made to better understand the relationship between KFD and Hashimoto's disease, particularly because of the suggested autoimmune etiology of KFD.

## Data Availability

All data generated or analysed during this study are included in this published article and its supplementary information files.

## Abbreviations

KFD: Kikuchi-Fujimoto disease
CBC: Complete blood count
CT: Computed tomography
SLE: Systemic Lupus Erythematosus
LN: Lymph node
